# A systematic review of the effectiveness of interventions to improve post-fracture investigation and management of patients at risk of osteoporosis

**DOI:** 10.1186/1748-5908-5-80

**Published:** 2010-10-22

**Authors:** Elizabeth A Little, Martin P Eccles

**Affiliations:** 1 Institute of Health and Society, Newcastle University, Baddiley-Clark Building, Richardson Road, Newcastle upon Tyne, NE2 4AX, UK

## Abstract

**Background:**

There is a large quality of care gap for patients with osteoporosis. As a fragility fracture is a strong indicator of underlying osteoporosis, it offers an ideal opportunity to initiate investigation and treatment. However, studies of post-fracture populations document screening and treatment rates below 20% in most settings. This is despite the fact that bone mineral density (BMD) scans are effective at identifying patients at high risk of fracture, and effective drug treatments are widely available. Effective interventions are required to remedy this incongruity in current practice.

**Methods:**

This study reviewed randomised controlled trials (RCT) involving fully qualified healthcare professionals caring for patients with a fragility fracture in all healthcare settings. Any intervention designed to modify the behaviour of healthcare professionals or implement a service delivery change was considered. The main outcomes were BMD scanning and osteoporosis treatment with anti-resorptive therapy. The electronic databases Medline and Embase were searched from 1994 to June 2010 to identify relevant articles in English. Post-intervention risk differences (RDs) were calculated for the main outcomes and any additional study primary outcomes; the trials were meta-analysed.

**Results:**

A total of 2814 potentially relevant articles were sifted; 18 were assessed in full text. Nine RCTs evaluating ten interventions met the inclusion criteria for the review. All were from North America. Four studies focused on patients with a hip fracture, three on fractures of the wrist/distal forearm, and two included several fracture sites consistent with a fragility fracture. All studies reported positive effects of the intervention for the main study outcomes of BMD scanning and osteoporosis treatment. For BMD scanning the overall risk ratio (95% CI) was 2.8 (2.16 to 3.64); the RD was 36% (21% to 50%). For treatment with anti-resorptive therapy the overall risk ratio (95% CI) was 2.48 (1.92 to 3.2); the RD was 20% (10% to 30%).

**Conclusions:**

All interventions produced positive effects on BMD scanning and osteoporosis treatment rates post-fracture. Despite sizeable increases, investigation and treatment rates remain sub-optimal. Long-term compliance with osteoporosis medications needs to be addressed, as the majority of studies reported treatment rates at six-month follow up only. Studies would be more informative if treatment criteria were defined *a priori *to facilitate understanding of whether patients were being treated appropriately and integrated economic analyses would be helpful for informing policy implementation decisions.

## Background

Osteoporosis is, 'a progressive systemic skeletal disease characterised by low bone mass and micro-architectural deterioration of bone tissue, with a consequent increase in bone fragility and susceptibility to fracture [1].' Osteoporosis can be diagnosed clinically (vertebral fracture in a 80 year old white female) or defined by a T-score of -2.5 standard deviations or lower on bone mineral density (BMD) scanning. It is well documented that there is a large quality of care gap for patients with this condition [2-11]. This has two main components: firstly a failure to make a diagnosis of osteoporosis, and secondly to manage the condition adequately once the diagnosis has been made.

A fragility fracture is 'a fracture caused by injury that would be insufficient to fracture normal bone: the result of reduced compressive and/or torsional strength of bone' [12]. It is a strong indicator of underlying osteoporosis, and it has been shown that adults who sustain a fracture are over 50% more likely to have another at a different anatomical site [13,14]. Therefore, a first fracture offers an ideal opportunity to initiate investigation and, if indicated, treatment for osteoporosis. However, studies of post-fracture populations document screening and treatment rates below 20% in most settings [5,8-11,15-20]. This is despite the fact that BMD scans are effective at identifying patients at high risk of fracture [21-23], and drug treatments have been shown to significantly reduce the rates of subsequent fragility fractures, even in individuals with advanced bone loss and prevalent fractures [24-30].

Although published evidence-based guidelines exist for the investigation and management of osteoporosis [31-35], the gap between accepted recommendations for osteoporosis care and current practice remains wide. The reasons for this are unclear, although several barriers have been suggested and explored [36-43]. Reported reasons include: lack of consensus as to who is responsible for initiating treatment; lack of awareness by patients and physicians of the treatment guidelines and efficacy of medications for osteoporosis following fragility fracture; and the adverse effects and high costs of medications. Recent studies have shown that 70% to 90% of PCPs wish to be more informed about the management of osteoporosis [39,41,42], and with PCPs assuming the prime responsibility for addressing osteoporosis over recent years, this is an ideal setting in which to implement change.

The aim of this review is to assess within randomised controlled trials (RCTs) the effectiveness of interventions to improve the investigation (BMD scanning) and management of osteoporosis (treatment with anti-resorptive therapy) in patients following a fragility fracture.

## Methods

### Criteria for considering studies for this review

#### Types of studies

This review focused on RCTs as they provide the least biased estimate of the effectiveness of an intervention.

#### Types of participants and settings

This review focused on fully qualified healthcare professionals of any discipline caring for patients with a fragility fracture. All healthcare settings were included, *i.e*., community, primary, secondary, and tertiary care.

#### Types of interventions

This review focused on any intervention or combination of interventions designed to improve the investigation and management of osteoporosis following fragility fracture by modifying the behaviour of healthcare professionals or implementing a service delivery change, with usual care as comparator.

### Outcome measures

The main review outcomes of interest were BMD scanning and osteoporosis treatment with anti-resorptive therapy. Other outcomes considered were: diagnosis of osteoporosis, prescribing of calcium and vitamin D, and economic variables. A study was required to report on at least one of the main review outcomes to be considered for this review.

### Search methods for identification of studies

The electronic databases Medline and Embase were searched from 1994 (reflecting the introduction of BMD scanning and use of anti-resorptive medications such as the bisphosphonates into practice) to June 2010. The search strategy incorporated the Cochrane RCT sensitivity maximising filter combined with selected MeSH terms and free text terms related to interventions to improve investigation and management of osteoporosis following fragility fracture (see Additional File 1 for the search strategy used in full). The search was limited to English language articles.

In addition to the electronic searches, the reference lists of relevant studies were hand searched to identify any further relevant studies, and the following were contacted to enquire about any additional published or unpublished data relevant to this review: National Osteoporosis Society (UK), National Osteoporosis Foundation (US), International Osteoporosis Foundation, and experts in the field.

### Data collection and analysis

#### Selection of studies

One review author (EAL) screened all titles and abstracts of retrieved studies in Endnote. If a study met the initial selection criteria or its eligibility could not be determined from the title and abstract, the full text was retrieved. Both review authors then independently assessed the full text for inclusion status, and any disagreements were resolved through discussion.

#### Data extraction and management

EAL undertook data abstraction of each of the included studies using a modified Cochrane Effective Practice and Organisation of Care (EPOC) Data Collection Checklist. The data extraction form was pilot tested on one included study. Data was extracted on study design, study objectives, participants, instrument reliability and validity, type of interventions, sample size, statistical power, primary and secondary study findings, statistical tests used, and associated statistical and clinical significance. MPE independently assessed the data extracted and conclusions drawn.

#### Assessment of risk of bias in included studies

We used The Cochrane Collaboration's tool for assessing risk of bias on six standard criteria: adequate sequence generation, concealment of allocation, blinded or objective assessment of primary outcome(s), adequately addressed incomplete outcome data, free from selective reporting, and free of other risk of bias [44]. We used three additional criteria specified by the EPOC Review Group: similar baseline characteristics, similar baseline outcome measures, and adequate protection against contamination [45]. No studies were excluded because of poor methodological quality.

#### Measures of treatment effect

We report the main results for each study in natural units extracted from the results presented in articles. Only the main review outcomes, as well as additional study primary outcomes are reported in full. We had planned to report adjusted risk differences for the review outcomes that adjust for baseline compliance. However, clinicians were unlikely to test those who have been tested or treat those who were already treated for osteoporosis, making the notion of baseline compliance with the review outcomes less meaningful. Therefore, post-intervention risk differences (RD) were calculated and reported instead. For a study to be included in the review, it had to report actual numbers of patients receiving a BMD scan or anti-resorptive treatment for osteoporosis following fragility fracture to enable the post-intervention RD to be calculated (one study was rejected on these grounds). As an overall summary measure of effectiveness, the risk ratio and RDs were calculated using meta-analysis for both of the main outcomes of the review.

## Results

### Selection of studies for inclusion

**Figure 1:**
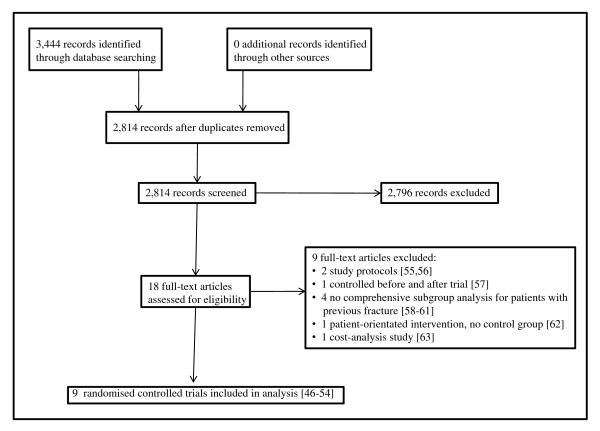
**Study Flowchart**.

Figure 1 describes the process from searching to study inclusion. Searches of the electronic databases to June 2010 yielded a total of 2,814 potentially relevant articles (following de-duplication). Following review of titles and abstracts, we obtained 18 articles for assessment in full text. Nine RCTs met the inclusion criteria for the review [46-54], with nine being excluded for the reasons detailed in Figure 1[55-63]. No additional potential studies were identified through hand-searching reference lists of articles, or by contacting experts in the field or osteoporosis foundations/societies.

### Characteristics of study design and setting

Table 1 describes the included studies. There were eight two-arm RCTs [46,48-54] and one three-arm RCT [47]. Five of the studies were conducted in the US [46,47,50,53,54] and four in Canada [48,49,51,52]. Four of the studies were set in tertiary care university hospitals or medical centres [46,48,53,54], with the intervention being targeted at primary care physicians (PCPs) in three of them [46,48,54]. Two studies were set solely in primary care [47,50]. The remaining three studies were set in hospital [49,51,52] with the PCP being the target of the intervention in two of them [51,52]. The unit of allocation was the patient in seven [46-49,52-54], the physician in one [50], and the family practice in one [51]. It was not possible to produce an overall number of sites and healthcare professionals; the studies included 3,302 patients.

**Table 1:** Characteristics of included studies

Reference	Setting	Design	Trial subjects	Inclusions	Exclusions	Content and method of delivery of intervention	Control group	Comments
Gardner 2005 [46]	One tertiary care university medical centre; primary care; New York, USA.	Two arm RCT; patient randomised. No power calculation reported. F/U period: six months.	**Clinicians** PCPs* (further details not reported). **Patients** N = 80. Mean age: 82 years. 78% female.	Low energy hip fracture.	Antiresorptive medication use, under 65 years, alcoholism, dementia.	**Content** 15 mins patient education; five questions to take to PCP regarding investigation, diagnosis and management of osteoporosis; patient reminder at six weeks post-op. **Delivery** Visit by clinical research coordinator during hospitalization; printed copy of questions; phone call.	Prior to discharge, patients given two page pamphlet on fall prevention based on a National Osteoporosis Foundation publication.	

Feldstein 2006 [47]	One Pacific Northwest non-profit health maintenance organization (HMO) involving 15 primary clinics; USA.	Three arm RCT; patient randomised. Power calculation reported and sufficient numbers recruited. F/U period: six months.	**Clinicians** 159 PCPs. **Patients** N = 327. Age range = 50-89 years. 100% female.	Individuals aged 50 to 89 who had been HMO members for at least 12 months and sustained a study defined fracture (any clinical fracture except skull, facial, finger, toe, ankle or any open fracture).	Previous BMD scan/osteoporosis treatment, malignancy, chronic renal failure, organ transplant, cirrhosis, dementia, men, nursing home residents, no address, no primary care provider, research centre employees.	**Intervention one**: **Content** Physician alert and education. **Delivery** Patient-specific electronic medical record (EMR) in-basket message from chairman of the osteoporosis quality-improvement committee; internal and external guideline resources; second message sent at three months if no investigation/treatment carried out. **Intervention two**: **Content** Physician alert and education; patient reminder and education copied to PCP. **Delivery** Patient-specific EMR in-basket message as above with copy of patient reminder; printed educational materials in advisory letter to patient.	Usual care - if patient is hospitalized for a fracture, the PCP receives a copy of the discharge summary and the patient is followed-up by orthopaedists in a fracture clinic.	Except for exclusion of open fractures, no attempt made to distinguish between fractures that resulted from high force as data not reliably available electronically.

Davis 2007 [48]	One tertiary care university hospital; primary care; Vancouver, Canada.	Two arm RCT; patient randomised. Power calculation reported. F/U period: six months.	**Clinicians** PCPs (further details not reported). **Patients** N = 48. Mean age: 82.6 years (control), 80.4 years. (intervention) 71% female.	All women and men ≥ 60 years residing in Vancouver admitted with a minimal trauma hip fracture.	On osteoporosis treatment, dementia/cognitive impairment, unable to communicate in English, severe medical pathology (e.g. cancer, chronic renal failure).	**Content** Patient education and advice to visit PCP for further investigation; physician alert. **Delivery** Osteoporosis information; letter for patient to take to PCP from orthopaedic surgeon.	Usual care for the fracture and a phone call at three months (general health inquiry) and 6 months to determine whether osteoporosis investigation and treatment had occurred.	Minimal trauma defined as falling from a standing height or less. Power calculation required sample size of 44. 48 subjects recruited but 20 in control group and 28 in intervention group. No explanation for uneven numbers between groups reported.

Majumdar 2007 [49]	Three hospitals in Capital Health System; Edmonton, Alberta, Canada.	Two arm RCT; patient randomised. Power calculation reported and sufficient numbers recruited. F/U period: six months.	**Clinicians** One case-manager (registered nurse), one study physician. **Patients** N = 220. Median age: 74 years. 60% female.	Community-dwelling patients ≥ 50 years with hip fracture undergoing surgical fixation with no contraindications to bisphosphonates and able to provide (or have a proxy provide) informed consent.	Delirium, dementia, on osteoporosis treatment, pathologic fractures, patients in nursing homes or long-term care facilities.	**Content** Usual care; patient education; outpatient BMD test; prescription for bisphosphonates for patients with low bone mass; communication to PCPs regarding results and treatment plans. **Delivery** Case-manager - provided one-on-one counselling; arranged BMD test; obtained prescription from study physician to be dispensed by local community pharmacy.	Study personnel provided counselling about fall prevention and intake of calcium and vitamin D; educational materials from osteoporosis Canada provided and patients asked to discuss the material with their PCP.	Canadian guidelines recommended pharmacologic osteoporosis therapy in patients with a fragility fracture after age 50 years or menopause and a BMD T score ≤ -1.5. Patients in control group received more education and study-related attention than true usual care as practiced in most Canadian or US centres.

Solomon 2007 [50]	Primary care (patients all beneficiaries of HBCBSNJ^Δ ^health care insurer); New Jersey, USA.	Two arm cluster RCT; physician randomised (provided at least four patients per physician). Analysis adjusted for clustering. No power calculation reported. F/U period: 10 months.	**Clinicians** 434 PCPs. Mean age: 50 years. 17% female. **Patients** N = 1973 (229 with fractures). Mean age: 69 years (control), 68 years (intervention). 92% female.	HBCBSNJ beneficiaries who had at least two years of enrolment and a prescription drug benefit; required to have filed at least one prescription claim in each of the two baseline years; age ≥ 45 years; prior fracture of hip, spine, forearm or humerus.	Previous BMD scan or prescription for osteoporosis medication during baseline 26 months; patients whose PCP had < four eligible patients at risk for osteoporosis.	**Content** Physician education; physician alert; patient education; patient invitation to attend BMD scan. **Delivery** One-on-one educational visit with PCP conducted by specially trained pharmacists who work with HBCBSNJ as physician educators; continuing medical education (CME) program; list of at-risk patients given to PCP and discussed at meeting; printed educational materials and letter from HBCBSNJ to patient; automated phone call invitation for BMD scan.	No description, assumed usual care.	Figures for subgroup of patients with prior fracture included in review taken from baseline characteristics of wider study population. The study paid for doctors to apply for CME credit if they completed a post-visit test.

Cranney 2008 [51]	Emergency departments or fracture clinics of five hospitals (two of which were teaching hospitals); 119 primary care practices; Ontario, Canada.	Two arm cluster RCT; family practice randomised. Analysis adjusted for clustering. Power calculation reported and sufficient numbers recruited. F/U period: six months.	**Clinicians** 174 PCPs. 55% female. 54 practiced in rural settings. **Patients** N = 270. Mean age: 69.8 years (control), 68.1 years (intervention). 100% female.	Family practices in Kingston, Ontario and the surrounding southeastern Ontario region drawn from the Canadian Medical Association directory; post-menopausal women who had sustained a wrist fracture (confirmed by x-ray).	Osteoporosis medication use, traumatic wrist fracture, unable to communicate in English or unable to give consent.	**Content** Physician alert; physician education; patient reminder recommending F/U visit with PCP; patient education. **Delivery** Personalised letter mailed to PCP by research coordinator at two weeks and two months post-fracture; two page educational tool and treatment algorithm from Osteoporosis Canada's clinical practice guidelines; mailed patient reminder letter at two weeks and two months post-fracture; educational booklet.	Usual care. Patients and PCPs were not sent any communication until trial completed.	

Majumdar 2008 [52]	Two emergency departments and two fracture clinics, Capital Health; primary care; Edmonton, Alberta, Canada.	Two arm RCT; patient randomised. Power calculation reported and sufficient numbers recruited. F/U period: six months.	**Clinicians** 266 PCPs. **Patients** N = 272. Median age: 60 years. 77% female.	Age ≥ 50 years and any distal forearm fracture, regardless of cause.	Bisphosphonate use, unable or unwilling to provide informed consent, no fixed address, residing outside Capital Health region, residing in a long-term care facility.	**Content** Patient education and advice to discuss osteoporosis with PCP; patient-specific reminders to PCPs; physician education. **Delivery** Phone counselling session to patients by experienced registered nurse; physician reminder sent by fax or mail; evidence based treatment guidelines endorsed by opinion leaders sent to PCPs.	Given Osteoporosis Canada pamphlet and encouraged to discuss with PCP, second copy mailed to patient. PCPs routinely notified that their patients had been treated for a wrist fracture and informed of F/U plans and appointment.	

Miki 2008 [53]	One tertiary care university medical centre, inpatient and outpatient clinic; Connecticut, USA.	Two arm RCT; patient randomised. Power calculation reported. F/U period: six months	**Clinicians** One male orthopaedic surgeon. **Patients** N = 62. Mean age: 79.2 years. 71% female.	All English-speaking patients admitted with low-energy hip fracture.	Osteoporosis medication use, pathologic fracture.	**Content** Patient education; osteoporosis evaluation; calcium and vitamin D commenced; patient review and bisphosphonate commenced as appropriate; monitoring of adherence to medication and complications; transfer of responsibility for medication adherence and patient management to PCP after six months. **Delivery** 15 mins education to patient and families whilst in hospital from one of the investigators; inpatient blood tests and BMD scan; F/U outpatient orthopaedic clinic appointment between two weeks and one month post-op; phone call to patient or clinic visit at two and six months.	15 mins education on hip fractures, fracture prevention and osteoporosis from one of the investigators; advised to see PCP for osteoporosis evaluation; commenced on calcium and vitamin D.	Trial stopped following interim analysis before pre-defined sample size reached due to ethical reasons.

Rozental 2008 [54]	One university tertiary care centre, orthopaedic outpatient clinic; primary care; Boston, USA.	Two arm RCT; patient randomised. Power calculation reported and sufficient numbers recruited. F/U period: six months.	**Clinicians** PCPs, orthopaedic surgeons (further details not reported). **Patients** N = 50. Mean age: 65 years. 92% female	Women > 50 years or men > 65 years; fragility fracture of distal part of radius.	High energy trauma, BMD scan within two years of fracture, current HRT or antiresorptive medication use.	**Intervention one**: **Content** BMD scan with results forwarded to PCP. **Delivery** Scan ordered by orthopaedic surgeon during patient's initial office visit for fracture care; results forwarded by mail and email to PCP.	**Intervention two**: Letter sent by email and mail to PCP outlining national guidelines for evaluating and treating osteoporosis after fragility fracture; the guidelines included ordering a BMD scan within six months of injury.	Intervention two considered to be close enough to usual care to use as a control group. Fragility fracture defined as those resulting from a standing height or less.

* PCPs = Primary care physicians

^Δ ^HBCBSNJ = Horizon Blue Cross Blue Shield of New Jersey

Four studies focused on patients with a hip fracture [46,48,49,53], three on fractures of the wrist/distal forearm [51,52,54], and two included several fracture sites consistent with a fragility fracture [47,50]. One study specified that the fracture was a fragility fracture [54], and four that the fracture was low energy/minimal trauma [46,48,51,53], but the remaining four studies did not discriminate by mechanism of injury [47,49,50,52].

### Types of intervention

The intervention (content and method of delivery) and the care delivered to the control groups are described in Table 1. Two of the interventions were directed at the PCP through patient education [46,48], one was an electronic medical record (EMR) reminder sent to the PCP [47], one included an EMR reminder to the PCP plus a patient reminder [47], three incorporated both PCP and patient education [50-52], and three were service delivery changes [49,53,54]. Two of the service delivery changes took the responsibility of investigating and treating the osteoporosis out of the hands of the PCP [49,53], and in one the investigation was carried out but the results were sent to the PCP to act on [54].

### Development of the intervention

With regards to the development of the intervention, only two studies reported consulting with the professional recipients [49,52]. Six studies reported the evidence base for the intervention [46,47,49-52]. Consumer involvement was not reported by any of the studies. In four of the studies, the authors report specific barriers to change that the intervention was tailored to address [46,49,51,53].

### Risk of bias in included studies

The risk of bias in included studies is reported in Table 2. Six trials reported adequate sequence generation, four reported adequate concealment of allocation, and four reported either adequately blinded or objective assessment of primary outcome. All studies adequately addressed incomplete outcome data, but only for two studies did it appear that they were free from selective reporting. Seven studies were judged to be free from other biases; one of the other two studies was stopped early, reporting that it was deemed unethical to continue following an interim analysis. It is unclear if this interim analysis was pre-specified. Eight studies had similar baseline characteristics and all had similar baseline outcome measures (for treatment but not for BMD scanning). Three studies were judged to have adequate protection against contamination.

**Table 2:** Risk of bias of included studies

Reference	Adequate sequence generation	Concealment of allocation	Blinded or objective assessment of primary outcome(s)	Adequately addressed incomplete outcome data	Free from selective reporting	Free from other risk of bias	Similar baseline characteristics	Similar baseline outcome measures	Adequate protection against contamination
Gardner 2005 [46]	Yes	Unclear	Unclear	Yes	Unclear - protocol not published; trial registration number not reported.	Unclear - only approx. 20% of patients approached were included in the study. 40% of patients deemed eligible declined to enter study.	Unclear - not reported.	Yes for treatment; unclear for BMD scanning.	Unclear - patient randomised, PCPs not reported.

Feldstein 2006 [47]	Yes	Yes	Yes	Yes	Unclear - protocol not published; trial registration number not reported.	Yes	Yes	Yes	Unclear - patient randomised: 15 primary care clinics involved with 159 PCPs, average one to three patients per PCP.

Davis 2007 [48]	Yes	No	No	Yes	Unclear - protocol not published; trial registration number not reported.	Yes	Yes	Yes for treatment; unclear for BMD scanning.	Unclear - patient randomised, PCPs not reported.

Majumdar 2007 [49]	Yes	Yes	Yes	Yes	Yes for primary outcome; no for secondary outcomes.	Yes	Yes	Yes for treatment; unclear for BMD scanning.	Unclear - possibility of contamination if control and intervention patients on ward at same time.

Solomon 2007 [50]	Unclear	Unclear	Unclear	Yes	Unclear - protocol not published; trial registration number not reported.	Yes	Yes	Yes	Unclear - physician randomised but practices in which they worked not reported on.

Cranney 2008 [51]	Yes	Yes	Yes	Yes	Unclear - protocol not published; trial registration number not reported.	Yes	Yes	Yes	Yes

Majumdar 2008 [52]	Yes	Yes	Yes	Yes	Yes	Yes	Yes	Yes for treatment; unclear for BMD scanning.	Yes

Miki 2008 [53]	Unclear	Unclear	No	Yes	Unclear - protocol not published; trial registration number not reported.	Unclear - stopped early as deemed unethical to continue following interim analysis.	Yes	Yes	Yes

Rozental 2008 [54]	Unclear	Unclear	Unclear	Yes	Unclear - protocol not published; trial registration number not reported.	Yes	Yes	Yes for treatment; unclear for BMD scanning.	Unclear - patient randomised, PCPs not reported.

Although producing summary scores or categories across the various risk of bias criteria is not recommended, the results in Table 2 suggest that only one-third of the studies were likely to be at low risk of bias.

### Effects of interventions

**Figure 2:**
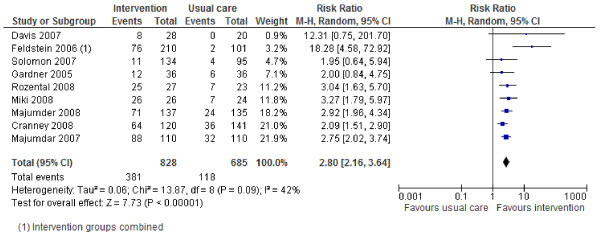
**Risk ratio for bone mineral density scanning (Mantel-Haenszel, random effects)**.

**Figure 3:**
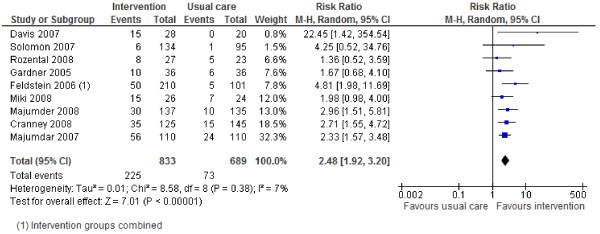
**Risk ratio for anti-resorptive drug treatment (Mantel-Haenszel, random effects)**.

**Figure 4:**
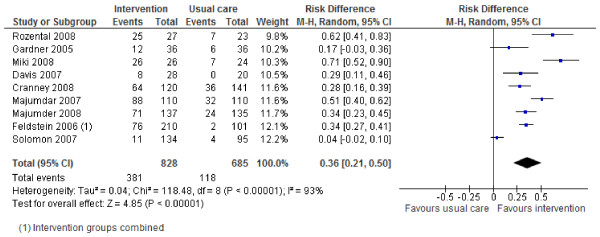
**Risk difference for bone mineral density scanning (Mantel-Haenszel, random effects)**.

**Figure 5:**
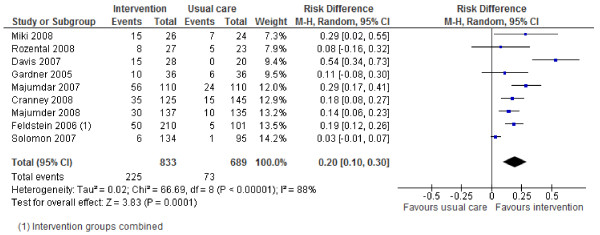
**Risk difference for anti-resorptive drug treatment (Mantel-Haenszel, random effects)**.

Review outcomes and study primary outcomes of included studies are reported in Table 3. With regards to the main review outcomes of BMD scanning and osteoporosis treatment with anti-resorptive therapy, all studies report positive effects of the interventions. The results are shown in Forest Plots in Figures 2 to 5. For BMD scanning the overall risk ratio (95% CI) was 2.8 (2.16 to 3.64) and there was a small to medium, non-significant amount of heterogeneity (I^2 ^42%); the RD was 36% (21% to 50%). For treatment with anti-resorptive therapy the overall risk ratio (95% CI) was 2.48 (1.92 to 3.2) and there was no heterogeneity (I^2 ^7%); the RD was 20% (10% to 30%). Funnel plots (Additional File 2) suggest some asymmetry, but there are too few studies to formally assess this.

**Table 3:** Reported study outcomes

Reference		Reported study outcomes			Osteoporosis medication use	Comments
Gardner 2005 [46]		**Control**	**Intervention**	**Post-intervention RD**^**Δ**^	**Drugs used**	
		N (%)	N (%)	(%)	Bisphosphonates.	
	BMD scan	6/36 (17)	12/36 (33)	17	**Data source for drug utilisation**	
	Osteoporosis treatment	6/36 (17)	10/36 (28)	11	Patient self-report.	

Feldstein 2006 [47]		**Control**	**Intervention**	**Post-intervention RD**	**Drugs used**	Secondary outcomes included regular physical activity, total caloric expenditure, total calcium intake and patient satisfaction.
		N (%)	N (%)	(%)	Bisphosphonate, calcitonin, selective estrogen receptor modulator, estrogen medication.	
	**EMR**				**Data source for drug utilisation**	
	BMD scan	2/101 (2)	40/101 (40)*	38	Electronically from outpatient pharmacy system.	
	Osteoporosis treatment	5/101 (5)	28/101 (28)*	23		No significant differences between the EMR and the EMR + patient reminder arm with respect to BMD scanning and osteoporosis treatment.
	**EMR + patient reminder**					
	BMD scan	2/101 (2)	36/109 (33)*	31		
	Osteoporosis treatment	5/101 (5)	22/109 (20)*	15		

Davis 2007 [48]		**Control**	**Intervention**	**Post-intervention RD**	**Drugs used**	4/20 (20%) of the control group and 11/28 (39%) of the intervention group received a diagnosis of osteoporosis but this difference was not significant.
		N (%)	N (%)	(%)	Bisphosphonates.	
	BMD scan	0/20 (0)	8/28 (29)*	29	**Data source for drug utilisation**	
	Osteoporosis treatment	0/20 (0)	15/28 (54)*	54	Patient self-report.	
	Calcium + vitamin D	6/20 (30)	11/28 (39)	9		
	Exercise prescription	0/20 (0)	9/28 (32)*	32		

Majumdar 2007 [49]		**Control**	**Intervention**	**Post-intervention RD**	**Drugs used**	Secondary outcomes included "appropriate care" (BMD testing with treatment if bone mass low), recurrent fractures, admissions to hospital and death.
		N (%)	N (%)	(%)	Bisphosphonates - alendronate or risedronate.	Of 120 who underwent BMD testing, 25 (21%) did not have low bone mass. Of the 95 patients with low bone mass, 41 (43%) had a T score at hip or spine between -1.5 and -2.5, and 54 (57%) had a T score of ≤ -2.5.
	BMD scan	32/110 (29)	88/110 (80)*	51	**Data source for drug utilisation**	
	Osteoporosis treatment	24/110 (22)	56/110 (51)*	29	Not reported.	

Solomon 2007 [50]		**Control**	**Intervention**	**Post-intervention RD**	**Drugs used**	Only results adjusted for baseline characteristics significant, unadjusted results insignificant.
		N (%)	N (%)	(%)	HRT, calcitonin, raloxifene, bisphosphonates, teriparatide.	
	BMD scan	4/95 (4)	11/134 (8)*	4	**Data source for drug utilisation**	
	Osteoporosis treatment	1/95 (1)	6/134 (4)	3	Health-care utilisation data.	

Cranney 2008 [51]		**Control**	**Intervention**	**Post-intervention RD**	**Drugs used**	38/141 (27%) of control patients and 43/120 (36%)of intervention patients received calcium or vitamin D. This difference was not statistically significant.
		N (%)	N (%)	(%)	One patient raloxifene, 49 bisphosphonates.	Results for osteoporosis treatment reported for all patients randomised (270) whereas BMD scanning and calcium or vitamin D only reported for those completing follow up (261).
	BMD scan	36/141 (26)	64/120 (53)*	28	**Data source for drug utilisation**	Secondary outcomes included discussion with PCP regarding osteoporosis and changes in the participant's knowledge of osteoporosis.
	Osteoporosis treatment	15/145 (10)	35/125 (28)*	18	Patient self-report.	Although baseline BMD scanning was reported, the ARD cannot be calculated as the numbers at baseline were different to those included in the analysis.

Majumdar 2008 [52]		**Control**	**Intervention**	**Post-intervention RD**	**Drugs used**	58/135 (43%) of control patients and 91/137 (66%) of intervention patients received calcium and vitamin D. This difference was statistically significant.
		N (%)	N (%)	(%)	Bisphosphonates.	Results adjusted for acid peptic disease, osteoarthritis, current smoking, calcium and vitamin D use as significant differences found between intervention and control groups for these 5 variables.
	BMD scan	24/135 (18)	71/137 (52)*	34	**Data source for drug utilisation**	Secondary outcomes included "appropriate care" and quality of life.
	Osteoporosis treatment	10/135 (7)	30/137 (22)*	14	Patient self-report confirmed through dispensing records of local pharmacies.	Of the 95 patients who underwent BMD testing, 27 (28%) had normal bone mass, 49 (52%) had osteopenia (T score -1.0 to - 2.5), and 19 (20%) had osteoporosis (T score ≤ -2.5) at either the hip or spine.

Miki 2008 [53]		**Control**	**Intervention**	**Post-intervention RD**	**Drugs used**	No p-value given for difference in BMD scanning between groups.
		N (%)	N (%)	(%)	One patient calcitonin nasal spray, 21 bisphosphonates.	For those starting osteoporosis treatment the post-intervention RD was 44% but one patient in the control group and five in the intervention group stopped before six months.
	BMD scan	7/24 (29)	26/26 (100)	71	**Data source for drug utilisation**	In the intervention group, 38% of those receiving treatment for osteoporosis had at least one T score of less than -2.5.
	Osteoporosis treatment	7/24 (29)	15/26 (58)*	29	Patient self-report.	Although baseline BMD scanning was reported, the ARD cannot be calculated as the numbers at baseline were different to those included in the analysis.
						Secondary outcomes included new fracture during the six month follow up period.

Rozental 2008 [54]		**Control (Intervention two)**	**Intervention One**	**Post-intervention RD**	**Drugs used**	Intervention two sufficiently close to usual care as to be considered as the control group.
		N (%)	N (%)	(%)	One teriparatide, one calcitonin, 11 bisphosphonates.	
	BMD scan	7/23 (30)	25/27 (93)*	62	**Data source for drug utilisation**	4/23 (17%) of control patients and 15/27 (56%) of intervention patients received calcium and vitamin D. This difference was not statistically significant.
	Osteoporosis treatment	5/23 (22)	8/27 (30)	8	Patient self-report and review of medical records.	Calcium and vitamin D counted as osteoporosis treatment in original paper so post-intervention RD reported here substantially less (8% c.f. 48%). There was a significant difference between groups when calcium and vitamin D were included as osteoporosis treatment but no p-values reported with these excluded.
	Treatment discussed with PCP	8/23 (35)	24/27 (89)*	54		2/23 (9%) of patients in the control group and 9/27 (33%) of patients in the intervention group received a diagnosis of osteoporosis.

*statistically significant difference between groups (p < 0.05).

ΔRD = risk difference.

### Other outcomes

Calcium and vitamin D use was reported in four studies [48,51,52,54], but there was only a statistically significant difference between control and intervention group in one (43% versus 66%) [52]. Two studies reported rates of osteoporosis diagnosis but did not specify what constituted this diagnosis [48,54]. One study reported the percentage of patients undergoing BMD scan given a diagnosis of osteoporosis (T score ≤-2.5) [52]. None of the studies reported concurrently economic outcomes, but one trial group [49] subsequently published a separate cost analysis of a case manager intervention that suggested that the intervention was cost saving [63]. Two studies reported on 'appropriate care' [49,52].

Two studies reported primary outcomes other than those addressed by this review [48,54]. One looked at whether or not participants were prescribed exercise as recommended by the 2002 Canadian Medical Association Osteoporosis Clinical Practice Guidelines [48]. They found a statistically significant difference between control and intervention groups with a post-intervention RD of 32%. The other study looked at whether or not osteoporosis had been discussed with the PCP and also found a statistically significant difference between intervention and control groups with a post-intervention RD of 54% [54].

## Discussion

We reviewed nine RCTs assessing the effectiveness of a variety of interventions to improve the investigation and management of osteoporosis following fragility fracture. For both of the review main outcomes, BMD scanning and osteoporosis treatment with anti-resorptive therapy, all studies reported a positive effect of the intervention with an overall 36% absolute increase in scanning rates and a 20% absolute increase in treatment rates. Such effects are clearly important. These results are broadly in agreement with other recent reviews [64] though this had a slightly different focus (patients at risk of osteoporosis) and so included different studies.

Whilst all studies reported positive effects, they have a number of constraints that limit their informativeness. All of the studies were conducted in North America. This may limit their generalisability to other countries and healthcare systems. Although we pooled the results of the studies (reflecting their homogeneity of patient groups and settings), there were still a range of types of interventions and it is not possible to say that any one intervention was more effective than any other. With relatively low levels of control group performance, the results suggest that any intervention is likely to be more effective than usual care; it is not clear what would happen in situations with higher rates of baseline performance. In relation to this, it is relevant to point out that this is a relatively recent body of literature with the oldest trial reporting in 2005. In future it is likely that studies will be addressing improved levels of baseline performance. In some studies it was not clear whether or not treatment was appropriately linked to BMD scanning result, with at least one study apparently reporting treatment on BMD scan values that would not result in treatment in the UK NHS [53]. Whilst it would be expected that most patients should receive a BMD scan post-fracture, the majority of studies failed to report treatment criteria *a priori *making it difficult to interpret treatment rates. One trial group reported an outcome of guideline concordant 'appropriate care' [49,52]. This was defined as a BMD test performed and osteoporosis treatment provided to those with low bone mass; they then defined low bone mass according to current guidelines. This maximises our understanding of the data by considering those patients who were appropriately not treated. In the 2007 study, the osteoporosis treatment rate in the intervention group was 51%, yet the rate of 'appropriate care' was 67% [49]. Thus, 16% of patients did not receive osteoporosis treatment following BMD scan because they were not eligible for it. Nevertheless, this study also highlights the fact that despite the overall positive effects of the interventions, none of the studies produced maximal rates of investigation and treatment. In this particular study, 33% of patients did not receive appropriate care.

All of the studies reported treatment rates at six months follow-up, except for one that extended to 10 months [50]. Long-term compliance with osteoporosis medications is not addressed and is something that will need to be considered in future studies.

We assessed all studies for their risk of bias, and five of the nine included studies had multiple unknown criteria in Table 2[46,48,50,53,54] It is unclear whether or not these studies were actually at an increased risk of bias or if this assessment was a consequence of poor reporting; however, they tended to be smaller and to report more uncertain results. It is not possible to exclude publication bias. The funnel plots suggested the possible absence of larger, less positive studies, but given the number of included trials this can only be a subjective judgement.

The rationale for the interventions used was often unclear. While four studies reported that the intervention was tailored to identified barriers [46,49,51,53], the other five did not. In addition, from some of the descriptions given it was difficult to extract sufficient detail to be confident that the interventions were being described in a way that would make them replicable [46-48,50,51,53]. It was also difficult to disentangle what the investigators felt was the content of their intervention (the active ingredients; *e.g*., persuasive communication) from the method that they chose to deliver it (printed educational materials). Such distinction is important in order to promote greater clarity in the description of interventions. Recent reporting guidelines have suggested that this will constrain scientific replication and limit the subsequent introduction of successful interventions [65].

Investigators should be considering the economic implications of their interventions and do not, on the basis of this review appear to be doing so; no study concurrently reported an integrated economic analysis of the intervention. However, one trial group subsequently reported an economic analysis of their case-manager intervention that suggested the intervention was cost saving [63].

The review had some limitations. Only articles published in English were considered and only two electronic databases were searched (Medline and Embase). However, it is reassuring that examination outside of the review of Web of Science, OVID Evidence Based Reviews and Cochrane (to June 2010) by one of this manuscript's reviewers identified no additional eligible studies (S. Majumdar, personal communication). Rather than having full duplication of all activities, one author (EAL) sifted the results of the search for included studies, although both authors assessed the eligibility of the 18 articles in which the full text was retrieved. One author (EAL) abstracted data from the included studies; this was checked by the second author.

## Summary

All interventions demonstrated a positive effect on BMD scanning and osteoporosis treatment post-fracture, regardless of healthcare setting, patient population, and type of intervention. Despite this, only one of the studies reported maximal investigation rates (all patients investigated) and none reported maximal treatment rates. Follow-up did not extend beyond 10 months in any of the studies; the issue of long-term compliance with osteoporosis medications will need to be addressed in future studies. To aid interpretation of results, study authors should report treatment criteria *a priori *and a measure of appropriate care. Integrated economic analyses would be helpful when considering widespread implementation.

## Conflicts of interests

MPE is Co-Editor in Chief of Implementation Science. All editorial decisions on this manuscript were made by another editor.

## Authors' contributions

MPE conceived the idea of the review. EAL wrote and conducted the searches and sifted the results. Both authors assessed the included and excluded studies. EAL led the writing of the manuscript. Both authors approved the final version.

## Supplementary Material

Additional file 1 **Search Strategy**. Click here for file

Additional file 2 Funnel plot of outcome: Bone Mineral Density scanning, Mantel-Haenszel random effects model; Funnel plot of outcome: Osteoporosis treatment, Mantel-Haenszel, random effects model. Click here for file
