# Supplementary material for: A systematic review of the effectiveness of interventions to improve post-fracture investigation and management of patients at risk of osteoporosis
Source: Implement Sci. 2010 Oct 22;5:80. doi: 10.1186/1748-5908-5-80 (PMC2988064; doi:10.1186/1748-5908-5-80)
Supplement: Additional file 1 — Search Strategy. [file 1748-5908-5-80-S1.DOC]

**Additional File 1: Search Strategy**

Medline/Embase to June 2010.

1. randomized controlled trial.pt.
2. controlled clinical trial.pt.
3. randomized.ab.
4. placebo.ab.
5. drug therapy.fs.
6. randomly.ab.
7. trial.ab.
8. groups.ab.
9. 1 or 2 or 3 or 4 or 5 or 6 or 7 or 8
10. exp animals / not humans.sh.
11. 9 not 10
12. exp Osteoporosis/
13. osteoporo$.mp.
14. 12 or 13
15. fragility fracture.mp. or exp Fractures, Bone/
16. fracture$.mp.
17. exp Diagnosis/ or diagnos$.mp.
18. management.mp. or Disease Management/ or exp Patient Care Management/
19. 15 or 16
20. 17 or 18
21. 11 and 14 and 19 and 20
22. Limit 21 to (English language and humans and yr = 1994 – current)
