# Supplementary material for: A systematic review of the effectiveness of interventions to improve post-fracture investigation and management of patients at risk of osteoporosis
Source: Implement Sci. 2010 Oct 22;5:80. doi: 10.1186/1748-5908-5-80 (PMC2988064; doi:10.1186/1748-5908-5-80)

Funnel plot of outcome: Bone Mineral Density scanning, Mantel-Haenszel random effects model


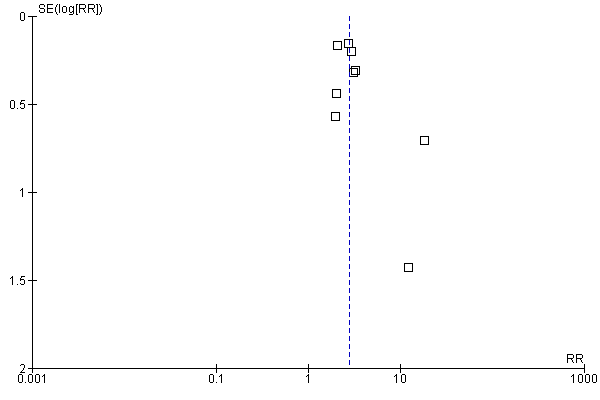


Funnel plot of outcome: Osteoporosis treatment, Mantel-Haenszel , random effects model.


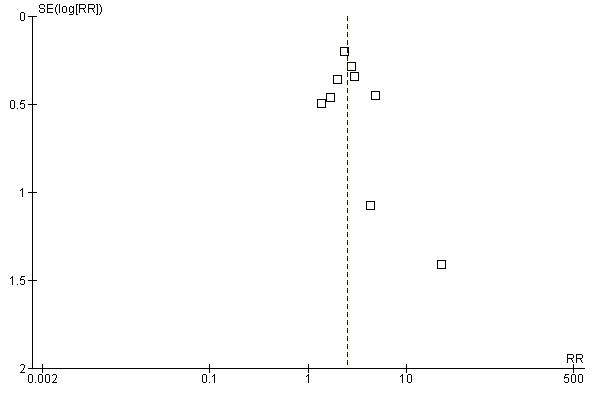

Supplement: Additional file 2 — Funnel plot of outcome: Bone Mineral Density scanning, Mantel-Haenszel random effects model; Funnel plot of outcome: Osteoporosis treatment, Mantel-Haenszel, random effects model. [file 1748-5908-5-80-S2.DOC]
